# Bayesian demography 250 years after Bayes

**DOI:** 10.1080/00324728.2015.1122826

**Published:** 2016-02-23

**Authors:** Jakub Bijak, John Bryant

**Affiliations:** ^a^ University of Southampton; ^b^ Statistics New Zealand; ^c^ University of Waikato

**Keywords:** Bayesian demography, Bayesian statistics, demographic methodology, population estimates and forecasts, statistical methods

## Abstract

Bayesian statistics offers an alternative to classical (frequentist) statistics. It is distinguished by its use of probability distributions to describe uncertain quantities, which leads to elegant solutions to many difficult statistical problems. Although Bayesian demography, like Bayesian statistics more generally, is around 250 years old, only recently has it begun to flourish. The aim of this paper is to review the achievements of Bayesian demography, address some misconceptions, and make the case for wider use of Bayesian methods in population studies. We focus on three applications: demographic forecasts, limited data, and highly structured or complex models. The key advantages of Bayesian methods are the ability to integrate information from multiple sources and to describe uncertainty coherently. Bayesian methods also allow for including additional (prior) information next to the data sample. As such, Bayesian approaches are complementary to many traditional methods, which can be productively re-expressed in Bayesian terms.

## 1. Introduction

The original paper of Thomas Bayes (1763) establishing the theorem that bears his name was presented to the Royal Society just over 250 years ago, two years after Bayes's death, by his friend Richard Price. Not long afterwards, Bayesian demography was born: in 1778, Pierre-Simon de Laplace, who later extended Bayes's theorem to a more general case, applied this method of inference to estimating sex ratios at birth for Paris and London (Laplace 1781 cited in Courgeau 2012). However, for about two centuries, Bayesian demography remained largely dormant. Only in recent decades has there been a revival of demographers’ interest in Bayesian methods, following the methodological and computational developments of Bayesian statistics. The area is currently growing fast, especially with the United Nations (UN) population projections becoming probabilistic—and Bayesian (Gerland et al. 2014).

The aim of this paper is to review the achievements of Bayesian demography, address some misconceptions about Bayesian approaches, and to make the case for more widespread use of Bayesian statistical methods, especially in currently underexplored areas of population studies. We review, synthesize, and evaluate three especially promising areas of application: demographic forecasts, problems with limited data, and highly structured and complex models. In addition to its pedagogical purposes, the paper contributes to the literature by offering suggestions for new, uncharted applications of Bayesian methods in demography. Throughout the paper we try to show how traditional demographic methods can be re-expressed in Bayesian terms, and how fruitful this approach can be.

For each of the three areas mentioned above, we discuss the main arguments put forward for the use of Bayesian methods, and illustrate them with selected examples from the demographic literature. Whilst the current review has inevitably omitted some particular pieces of work, we have attempted to cover all the main applications of contemporary Bayesian demography.

The paper is structured as follows. After this Introduction, in Section 2 we present the basic tenets of Bayesian statistics, with a particular focus on the practical areas of application that are relevant for contemporary demography, and critically evaluate some related misconceptions. We further explore three of these areas in more detail in Section 3, where we look at the following: population forecasting; population modelling for small samples, sparse or incomplete data; and the use of highly structured and complex models in demography. Finally, in Section 4, we discuss the future of Bayesian demography, its prospects and challenges.

## 2. Bayesian statistics in a nutshell

### 2.1. A brief history of the Bayesian approach

In contrast to the objectivist interpretation of probability, in the Bayesian approach, probability is typically interpreted as a representation of the subjective beliefs of the person drawing an inference. The objectivist approach, which is grounded on different philosophical premises, has been the dominant paradigm in statistics for most of the twentieth century. The most prominent school of thought, associated with Ronald A. Fisher, Jerzy Spława-Neyman, Egon S. Pearson, Karl Pearson, and others, linked probability with the frequencies of events under study (see Courgeau 2012 for details). The ‘frequentist’ school is mainly associated with methods for estimation and inference based purely on *likelihood*: the probability that a set of data was generated by a model with given parameters, treated as fixed, yet unknown. The frequentists were critical of the Bayesian perspective, mainly because of its explicit recourse to the notion of subjectivity.

Philosophical differences aside, computational demands also for a long time held back the practical application of Bayesian statistics. The Bayesian paradigm has made a slow but steady comeback to the statistical mainstream only since the 1970s, at first as a result of theoretical achievements (Savage [1954] 1972; Lindley 1972; for an overview, see Bernardo and Smith 2000). This process accelerated in the 1980s thanks to rapid developments in fast computing (Ntzoufras 2009). Owing to an increase in computing speed, and the development of Markov chain Monte Carlo (MCMC) methods (Geman and Geman 1984; Gelfand and Smith 1990), computations that were once difficult or impossible are now routine.

Currently, even though frequentist statistics still dominates in introductory statistical curricula, Bayesian inference and methods are becoming increasingly common in applied research in various fields, including demography and other related population sciences—from epidemiology (Broemeling 2013), to paleodemography (Caussinus and Courgeau 2010), to phylogenetics and historical linguistics (Greenhill and Gray 2009). Also now helping to popularize the methods are some useful introductory texts. Lynch (2007) has written an excellent introduction to a range of social science applications of Bayesian methods, and Hoff (2009) an important general textbook, which also includes many examples of application in the social sciences. For demographers, Bijak (2010) has produced a brief introduction to Bayesian statistics for population sciences, with a focus on migration.

**Figure 1:**
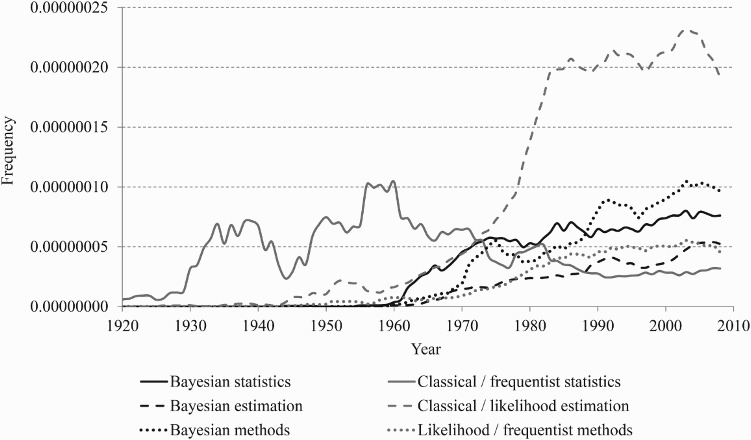
Frequencies for Bayesian and frequentist/likelihood search terms in Google books

*Note*: For caveats regarding the use of Google *N*grams in demography, see Bijak et al. (2014). *Source*: Google books *N*gram Viewer, http://books.google.com/ngrams, English corpus (accessed: 20 January 2014).

Some of these developments are visible in trends in relative frequencies of phrases (‘*N*grams’) used in publications—in this case, in the digital Google books collection. Figure 1 shows English-language examples of such trends, smoothed with five-term moving averages. The figure compares Bayesian statistics, estimation, and methods (black lines) with classical/frequentist/likelihood statistics, estimation, and methods (grey lines). Evidently, in digitized books, Bayesian statistics has been mentioned more frequently than classical/frequentist statistics since the 1980s. And even though classical/likelihood estimation is currently mentioned over four times more frequently in the Google books content, its relative frequency is stagnant, unlike its steadily increasing Bayesian counterpart. With respect to more practically oriented ‘methods’, the trends are even more favourable to the Bayesian approaches.

### 2.2. Bayesian theory: key aspects

The foundation of Bayesian analysis is Bayes's theorem (1763), which can be stated as follows:(1) 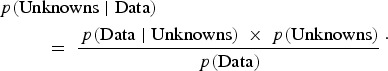



The ‘*p*’s in the equation denote probability distributions, that is, probabilities or probability density functions. The ‘unknowns’ may be abstract quantities such as regression coefficients, but may also be potentially observable quantities such as births that were missed by a registration system. The outcome from a Bayesian analysis is a posterior probability distribution for the unknowns, conditional on the data. The posterior distribution is obtained by multiplying the likelihood, *p*(Data | Unknowns), by the prior probability distribution, *p*(Unknowns), all divided by *p*(Data), the marginal likelihood, that is, the probability of obtaining a particular sample.

The fact that the outcome of an analysis is a probability distribution is an important advantage of Bayesian statistics. A probability distribution contains a great deal more information than the point estimate and standard error typically produced by a frequentist analysis. For instance, examination of the full distribution may reveal multiple modes, or show the probability mass to be concentrated in a certain area. But even more importantly, probability distributions can be summarized in ways that are more intuitively meaningful, both to laypeople and fellow scientists. It is, for instance, legitimate to interpret a Bayesian 95 per cent credible interval as having a 95 per cent probability of containing the true value. This is not the correct interpretation of a frequentist 95 per cent confidence interval. The ‘95 per cent’ from such a confidence interval refers to the proportion of hypothetical intervals that would contain the true value if the study were replicated many times. Converting a confidence interval into a statement about the data at hand requires further assumptions, like those described by a prior (Jaynes 1976; Howson and Urbach 2006).

In addition, it is straightforward to derive substantively meaningful quantities from a full probability distribution. It is, for instance, easy and natural to turn a joint probability distribution for age-specific mortality rates into a probability distribution for life expectancy. In contrast, turning point estimates and standard errors for age-specific mortality rates into a point estimate and standard error for life expectancy is technically more complicated and requires additional assumptions. Moreover, standard frequentist methods do not always produce the desired results when the numbers of deaths are small (Eayres and Williams 2004).

The likelihood component *p*(Data | Unknowns) in a Bayesian analysis is essentially the same as the likelihood in a frequentist analysis. In both cases it is a model for how the data were generated, given a set of parameters. A likelihood function might, for instance, state that births follow a Poisson distribution, with expected values that vary with age and population size. The marginal likelihood, *p*(Data), is fixed for any given data set and plays a minor role in most applications.

The prior, *p*(Unknowns), is distinctively Bayesian. It captures information about unknowns that is not contained in the data. An example of a prior could be a normal distribution with mean 1.8 and standard deviation 0.2, N(1.8, 0.2^2^), used to represent prior beliefs about a total fertility rate in a given developed country. Priors of this type, expressing quantitative statements about a parameter, are an important feature of applied Bayesian statistics. However, when data are abundant, it is more common for priors to have much less information content, and to be restricted to qualitative features of the data. For instance, a prior might state that a mortality profile is expected to rise monotonically with age, or that neighbouring regions are likely to be more similar than distant ones, or that household incomes can be treated as independent draws from the same distribution (Carlin 1992; Congdon 2010, Chapter 4). Indeed, priors may be even weaker than this, to the point where they are ‘non-informative’, and are dominated by the likelihood (Gelman et al. 2014, pp. 51–5). For such cases, the results of the analysis are often numerically close to their classical (frequentist) equivalents.

In modern Bayesian analyses, priors are often hierarchical. In other words, the parameters governing the priors are themselves given ‘hyper-priors’ governed by ‘hyper-parameters’. For instance, let 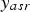 represent deaths of people of age *a* and sex *s* in region *r*; and let 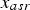 be the corresponding population at risk. Then, the age–sex–region specific mortality rates 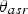
might be estimated using the following model, where the tilde ‘∼’ denotes ‘follows a distribution' and *M* stands for an arbitrary large number:
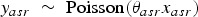


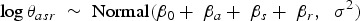


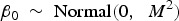


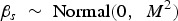


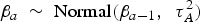

(2) 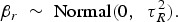



For the hyper-parameters, the following distributions can be assumed: 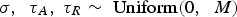.

The first equation in (2) is the likelihood. The second equation gives the prior for the mortality rates. The log-transformed rates are expected to follow normal distributions, with means varying by age, sex, and region. Each of the age, sex, and region effects in turn has a prior; for instance, the means for the age effects are expected to follow a random walk without drift. In this example, the intercept and sex terms, as well as all the standard deviations, are given vague (not very informative) priors.

As discussed further in Section 3, hierarchical models are an attractive way to model many population processes, and lead to sensible estimates when data are sparse. Frequentist versions of Bayesian hierarchical models exist, such as multilevel models or random-effects models. However, hierarchical models are particularly natural within the Bayesian framework because of the blurring of the distinction between observations and parameters, with both being treated as draws from probability distributions (Rubin 1984, p. 1154).

A computational shortcut when fitting a hierarchical model is to obtain point estimates for hyper-parameters using methods such as maximum likelihood, and to plug these estimates back into the model, treating them as known with certainty. This is referred to as an empirical Bayesian approach (e.g., Maritz 1970). The procedure needs to be used with caution because treating hyper-parameters as known when they are actually estimated, and using the same data twice—once to estimate hyper-parameters and again to estimate the remaining parameters—can lead to estimates that are spuriously precise. There are refinements of empirical Bayesian methods that address these concerns (Carlin and Louis 2009, Chapter 5), but the need to avoid estimating hyper-parameters within a full probabilistic model, and hence the motivation to use empirical Bayesian methods, is declining, owing to advances in computing power.

Finally, there is one set of applications where the marginal likelihood *p*(Data) does play an important role: Bayesian model selection and model averaging. Here the model specification itself is treated as an unknown (e.g., Raftery 1995). A set of alternative models is considered, and the posterior probability of selecting a particular model from this set, *p*(Model | Data), is also calculated by using Bayes's theorem. This approach requires setting prior probabilities for all the elements of the model space, *p*(Model), and providing the likelihood function, *p*(Data | Model), which is simply the marginal likelihood for the more standard single-model case. Calculating the marginal likelihood can be non-trivial and usually involves the use of sophisticated numerical algorithms, an overview of which can be found in Dellaportas et al. (2002). Once the posterior model probabilities are estimated, they can be used either to select the model with the highest data support, or to average the outcomes of different models, weighting them by these posterior probabilities. In the latter case, model uncertainty is explicitly included in the results.

### 2.3. Bayesian practice: computations and decisions

In a sense, once a Bayesian has specified a prior and a likelihood for a given data set, the modelling is finished. The result of the analysis—the posterior distribution—is now completely determined via Bayes's theorem. In practice, describing the implied posterior distribution can be challenging. Except in textbooks, the posterior distribution rarely takes a standard form such as a gamma or normal distribution, or other priors from the so-called ‘conjugate families’, for which convenient analytical solutions exist (see Gelman et al. 2014 for examples). More typically, the posterior can be obtained as a marginal distribution from the (multidimensional) joint probability distribution. Determining the shape of the posterior distribution, or even deriving some summary measures, was once the greatest practical obstacle to the use of applied Bayesian analysis.

Since the 1980s, many new methods for describing the posterior distribution have been developed. The dominant approach has been the MCMC (Gelfand and Smith 1990; Gilks et al. 1996; Gelman et al. 2014). MCMC yields an approximate sample from the posterior distribution. The analyst calculates summary statistics, such as means or intervals, for the approximate sample, and treats them as summary statistics for the true posterior distribution.

There are two key reasons why MCMC methods make tractable what would otherwise be intractable challenges to modelling. The first is that they allow the difficult problem of drawing a value from *p*(*θ*) to be replaced by the easier problem of generating a candidate value θ* and calculating *p*(θ*). The second is that they permit a divide-and-conquer approach: samples for the whole model can be constructed by successively sampling from each of the components. The main disadvantage of MCMC methods is that they can be computationally intensive and slow.

One area where Bayesian statistics lags behind frequentist statistics is user-friendly software for fitting standard models (for an overview see Wiśniowski's contribution in Bijak 2010). Fitting a basic Bayesian model typically requires more programming than an equivalent frequentist model. BUGS (Bayesian inference Using Gibbs Sampling) is the most mature general-purpose user-friendly package for Bayesian modelling (Lunn et al. 2009; Ntzoufras 2009), but it can struggle with large data sets or complex models. Bayesian computation is, however, an active field of research. New packages implementing general-purpose algorithms or specific techniques relevant to demography appear regularly on the ‘Bayesian Inference’ home page for the R programming language. The programming effort required for fitting a *non-standard* model, with features such as constraints or non-standard distributions, is often smaller for Bayesian models than it is for frequentist ones, thanks to the flexibility of MCMC (Gilks et al. 1996). Moreover, there are now many books such as Gelman and Hill (2006), Congdon (2010, 2014), Kruschke (2010), and Marin and Robert (2014) that use examples from the social sciences to teach Bayesian modelling and MCMC.

In coming years it seems likely that existing MCMC methods will cede ground to newer, faster techniques. Gelman et al. (2014), for instance, promote the software package STAN as a successor to BUGS. STAN combines standard MCMC with a related technique called Hamiltonian Monte Carlo (Neal 2011). Similarly, the statistical package INLA produces extremely fast and accurate approximations for a general class of Bayesian models (Rue et al. 2009).

In practical applications, the outcomes of Bayesian analysis have also the potential to serve as a basis for a formal decision support. Here, it is worth noting that the axiomatic foundations of Bayesian analysis are firmly rooted in statistical decision theory, and in the notions of utility or loss functions (see DeGroot [1970] 2004; and Bernardo and Smith 2000 for details; and Courgeau 2012 for a discussion). If the results of a Bayesian analysis can be combined with such utility or a loss function, reflecting the gains or losses from making particular decisions based on the unknown (estimated) quantities, the result is an elegant statistical system for decision-making (e.g., DeGroot [1970] 2004).

In brief, an optimal Bayesian decision for a given unknown quantity, described by a probability distribution, is one that maximizes the expected utility, or minimizes the expected loss. This approach allows the analyst to reduce whole probability distributions to point estimates, taking into account the respective costs or gains from over- and under-estimation, which need not be symmetric. Standard examples of such point estimates include the following: the median of the probability distribution for symmetric linear loss (utility) functions; quantiles for asymmetric linear functions; and the mean for quadratic function (DeGroot [1970] 2004). In practical applications, such utility or loss functions could measure the various outcomes—for instance, monetary—of decisions based on uncertain demographic parameters (for examples related to fiscal and other macroeconomic implications, sustainability of pension systems, or healthcare expenditure, see Alho et al. 2008).

### 2.4. Criticisms and misconceptions

Critics of Bayesian statistics have traditionally portrayed the need to specify a prior as a weakness of the Bayesian approach, on the grounds that it introduces an unacceptable element of subjectivity. This is an influential objection. It has, for instance, deterred the use of Bayesian methods by national statistical agencies, which are sensitive to any charge of subjectivity (Fienberg 2011; Pfeffermann 2013). Bayesians typically respond to such charges with three overlapping arguments: (i) some degree of subjectivity is unavoidable in any non-trivial statistical analysis; (ii) in many analyses, a wide range of sensible priors will lead to similar results; and (iii) in applications where it is impossible to avoid a substantial degree of subjectivity, documenting the subjective choices in the form of priors is a healthy and transparent practice.

A non-trivial statistical analysis—that is, an analysis involving imperfect data and complex relationships among variables—typically requires many choices that cannot be decided from the data alone. For instance, an analyst may have to choose between several possible error distributions, several plausible methods for dealing with missing values, or several possible sets of explanatory variables. These sorts of choices typically require knowledge about the process generating the data beyond what is contained in the data themselves. Such choices are necessary whether the analysis is Bayesian or frequentist, and arise in the specification of the likelihood as well as the prior. It is therefore misleading to say that priors make Bayesian analyses uniquely subjective.

A fundamental property of most Bayesian models is that, as the amount of data increases, the influence of the prior on the results declines. Moreover, with large, high-quality data sets and well-identified models, it becomes feasible to use vague or non-informative priors. Because demographers often use large, high-quality data sets, they can often avail themselves of such priors, and be confident that their results are not overly sensitive to the selection of the prior. This approach—‘objective Bayesianism’—allows the data to dominate the estimation process, and is argued to have desirable epistemological properties, especially from the point of view of the links with formal logic (Williamson 2013).

Besides the criticism of subjectivity, there are several examples of misconceptions related to particular demographic applications of Bayesian methods. The recent Bayesian population forecasts of the UN (Gerland et al. 2014) have been criticized as offering not much beyond simple extrapolations from the past into the future, devoid of expert input (see, e.g., the voices cited by Stukenberg 2014). This criticism is misplaced: Bayesian methods allow for the inclusion of expert knowledge formally and consistently in the models, through the prior distributions of the parameters. This has been done in the UN work by including informative priors intended to offer guidance on long-term asymptotic conditions in the absence of reliable empirical evidence (e.g., Raftery et al. 2013). The a priori information has been additionally augmented by the calibration of forecasting uncertainty based on the performance of the same model applied to various shortened data series from the past (see also the next section).

On the other hand, purely expert-based approaches either ignore other sources of information and uncertainty in the forecasts, such as past data series, or are overly reliant on the knowledge of experts. The use of the latter source on its own—without data—may pose problems—for a general discussion, see Lawrence et al. 2006; for a discussion of demographic examples of problems with purely expert-based assumptions and the related biases, see Oeppen and Vaupel 2002 or Keilman 2008. Such methods also usually do not attempt a formal calibration of uncertainty.

In short, Bayesian analysis does indeed contain explicit subjective elements, but it presents the subjectivities in a transparent way, and makes it possible to combine different types of information—data and expert knowledge—in a coherent manner. Moreover, the results are described by whole probability distributions, rather than just mean or median points, and as such convey much more information. An example of a practical misconception related to this aspect can be found in Brücker and Siliverstovs (2006), where Bayesian methods have been used simply as an alternative method of estimating point values of model parameters, which are presumably posterior means, although the prior and posterior distributions are not discussed explicitly.

Despite the subjectivity of some elements of the Bayesian approach, its transparency and coherence are increasingly appealing to statistical demographers in many areas of application, examples of which are reviewed and discussed next.

## 3. Key areas of demographic application

Despite the existence of Bayesian demographic studies in the eighteenth century (Laplace 1781), demography largely remained a deterministic exercise until the middle of the twentieth century. As argued by Courgeau (2012), this was largely due to the proliferation of census-based information. In particular, the availability of population-level figures has led to the problems of variability being largely ignored. Only after the Second World War did the challenges of uncertainty and probability begin to make a comeback within mainstream demography (Courgeau 2012). As mentioned by Alho (1999), there are examples of pioneering quasi-Bayesian population research from that period, notably the work of Leo Törnqvist's group on Finnish population forecasts (Hyppölä et al. 1949). A landmark paper by Alho and Spencer (1985) provided an overview of probabilistic population studies until the early 1980s, and also made a case for Bayesian analysis, labelled as a ‘more elegant’ solution to the population forecasting problem (Alho and Spencer 1985, p. 314).

Since the 1990s, Bayesian demography has been undergoing a revival, with a near-exponential growth in related research output, especially in the past decade. In this section, we examine the underlying driving forces behind this trend, for the three areas of application: forecasting, limited data, and highly structured and complex models. For these three areas, we review the motivations behind the use of Bayesian methods, and suggest some possible ways in which their applications may expand into new demographic territories over the course of the twenty-first century.

### 3.1. Forecasting

Population forecasts, and their less accountable cousins, population projections, are crucial for many areas of public policy, and for a variety of planning applications, in the public, private, and third sectors of the economy. That is why Booth (2006, p. 548) is right to refer to forecasting as ‘the public face of the [demographic] profession’. Partly in response to the demand for forecasts, and partly thanks to the adoption of methods from other disciplines of science, the past 30 years have witnessed very rapid methodological developments in this area (see Booth 2006 for an excellent overview).

As for successes in predicting populations, Xie (2000, p. 670) observes that demography has been ‘fundamental in forecasting future states of human societies  …  with a high degree of confidence’. This success can be attributed largely to the persistent regularities of some demographic processes, as well as to the deterministic nature of the underlying mechanism of population renewal. The latter manifests itself through population accounting and population momentum, with a lot of information being already embedded in age structures. These characteristics mark a clear contrast between quantitative demography's sphere of interest and those of other social sciences, and increase the predictability of populations, especially in the short term (Keyfitz 1981). Besides, as pointed out by Morgan and Lynch (2001), demography is heavily reliant on empirical data, which further strengthens the knowledge base of the forecasts it produces.

However, the issues with forecast errors are also well known, and have been extensively discussed, together with the need to communicate the errors to the forecast users (e.g., Keyfitz 1981; Keilman 1990; Alho and Spencer 2005). There are many sources of forecast uncertainty, and from the point of view of the forecasting process, six of these come especially to the fore: uncertainties in baseline data, model specification, model parameters, expert judgement on the assumptions, length of the forecast horizon, and the inherent randomness of the processes being forecast. This diversity calls for an integrated and coherent treatment of different types of uncertainty, ideally within a single approach.

With regard to forecast uncertainty, a natural advantage of Bayesian methods is that all different sources of error can be potentially embedded in a joint probabilistic forecasting model. Parameter uncertainty is reflected through prior distributions, which themselves can include the formally elicited uncertainty of expert opinion. Specification of the inherent randomness of the process forms a part of the model design, which can also include additional terms for baseline data errors. The issue of model specification can be addressed by adopting Bayesian model selection and averaging (Raftery 1995), as discussed in Section 2, and this has been done in several demographic applications to estimation (Murphy and Wang 2001) and forecasting (Bijak 2010; Abel et al. 2013a, 2013b).

This ability of Bayesian modelling to combine different uncertainties in a coherent manner also allows other approaches to population forecasting to be expressed in a Bayesian form, such as those based purely on expert opinion (Lutz et al. 2004), or purely data-driven time-series models (for an overview, see, e.g., Alho and Spencer 2005). These forecasts can then be interpreted as being conditional on the lack of uncertainty in the remaining aspects of the modelling process, such as parameters or models. In other words, these models could be seen as special cases of a fully Bayesian approach. Recently an attempt to reconcile the purely expert-based and Bayesian approaches has been undertaken by Billari et al. (2014), who proposed to give a Bayesian interpretation to expert-based predictions, and to treat expert inputs as data. This approach is subject to the same caveats as other expert-based propositions, since it does not formally incorporate the information carried by the time series of data.

Irrespective of a particular modelling approach, the outcome of the whole forecasting process in the Bayesian framework—a set of predictive distributions—not only offers a natural description of the overall uncertainty in the language of probabilities, but also follows directly from the joint statistical model and is very easy to derive and interpret (see, e.g., Bernardo and Smith 2000). The forecast uncertainty can be evaluated by assessing *ex post*, for example, based on truncated series or against external data, how well calibrated the probability distributions are. In its basic form, this exercise looks at whether the *ex post* distributions of forecast errors—obtained by comparing the forecasts with actual observations for a subset of data, such as a truncated time series—at least roughly match the ones predicted *ex ante* from the statistical model. In some cases, additional prior information gives an advantage in that respect: for example, assuming a priori low predictability of migration can often lead to better calibrated forecasts (Bijak 2010). At a more general level, Gneiting and Raftery (2007) have proposed several scoring rules which can be applied to produce a numerical summary of the calibration, as well as precision (*sharpness*) of the error distribution.

Furthermore, the joint probabilistic model can serve as a basis for deriving other conditional forecasts in a natural and straightforward manner, by assuming that certain parameters or processes are known without error. This can be interpreted as a probabilistic equivalent of ‘what-if’ scenarios (Bijak 2010). Moreover, under the Bayesian framework, predictions can be sequentially updated as soon as new information becomes available (Dawid 1984). Finally, probabilistic forecasts can serve as input for a formal decision analysis aimed at supporting the forecast users in their planning decisions, assuming that the loss functions can be at least approximately elicited from the decision-makers (Alho and Spencer 2005; Bijak 2010). This possibility remains still largely unexplored in demographic practice.

A further example of the usefulness of the Bayesian approach is age–period–cohort (APC) modelling, with clear forecasting applications, for example, by using models from the extended Lee and Carter (1992) family. The problem of the lack of identifiability of individual age, period, and cohort factors in a linear setting has long been known (see Fienberg 2013 for a recent overview and critique of some of the proposed approaches). Given the need for ‘resolving the APC dilemma using substantive judgment and knowledge’ (Fienberg 2013, p. 1982), Bayesian methods offer a transparent and flexible alternative, by including strong prior information on particular factors. Examples of Bayesian APC models are given by Nakamura (1986) and Berzuini et al. (1993), and in the dedicated software, ‘BAMP’, designed by Schmid and Knorr-Held (2007). It is worth noting that the APC analysis is useful both for forecasting and reconstructing past populations, as discussed in Section 3.3.

Apart from the pioneering work of Törnqvist's group in Finland (Hyppölä et al. 1949), the main developments in Bayesian population predictions began in the 1990s, with seminal papers by Bernardo and Muñoz (1993), on component-level population forecasts for Valencia, Spain, and by Daponte et al. (1997) on reconstructing the Iraqi Kurdish population under Saddam Hussein's regime. Bayesian demographic techniques have also been applied to non-human populations, with notable examples including bowhead whales (*Balaena mysticetus*, Raftery et al. 1995) and northern spotted owls (*Strix occidentalis caurina*, Clark 2003). Since the 1990s, both the methodological development of Bayesian methods and their areas of application have been expanding rapidly, and there has recently, especially since 2010, been a marked increase in the flow of contributions to Bayesian literature.

In this upsurge of interest, all three components of population change have received due attention from demographers applying Bayesian methods. For example, fertility forecasts have been presented by Tuljapurkar and Boe (1999) for the United States, and by Alkema et al. (2011) for the whole world, the latter in the context of ongoing work on the United Nations World Population Prospects (UN WPP). Recently, Schmertmann et al. (2014) proposed a method for predicting cohort fertility for countries featuring in the Human Fertility Database.

Bayesian mortality forecasts have been chiefly based on variants of the Lee and Carter (1992) bilinear model, examples of which include Czado et al. (2005) for France, Girosi and King (2008) for the United States, and Li (2014) for China and Taiwan. Girosi and King's book—despite a somewhat misleadingly broad title, since it is entirely devoted to mortality—addresses some of the shortcomings of the Lee and Carter model by analysing mortality by cause of death and incorporating covariates into the models. As alternatives, Lynch and Brown (2001) have compared three Bayesian models for compression and deceleration of mortality rates, based on the classical Gompertz model, logistic function, and a trigonometric transformation (arctangent). Lynch and Brown (2010), in turn, have extended Sullivan's method for reconstructing multistate life tables from period data, with potential direct applications in multistate projections or forecasts. Finally, Raftery et al. (2013) have produced forecasts of life expectancy for the whole world, also in the context of the UN WPP work; recently extended to a two-sex framework (Raftery et al. 2014).

For forecasting migration, a range of Bayesian time-series methods has been proposed, firstly by Gorbey et al. (1999) for flows between Australia and New Zealand; subsequently for a selection of European flows by Bijak (2010), and Bijak and Wiśniowski (2010); and then by Abel et al. (2013a) for environmental migration to the United Kingdom. Recently, Wis´niowski et al. (2014) have forecast Scottish migration after the 2014 referendum on independence, using a probabilistic mixture of two sets of forecasts, conditional on the referendum outcome. Given the high level of uncertainty and paucity of data on migration flows, many of these forecasts were making full use of informative priors, often based on explicitly expressed expert opinion. Finally, for other forms of mobility, examples include Congdon's (2000) Bayesian forecasts of patient flows to hospitals.

Recent years have seen several other examples of coherent Bayesian forecasts of whole populations, combining the predictions made for individual demographic components. Abel et al. (2013b) have provided a tutorial for overall time series of fertility, mortality, and migration for the United Kingdom, without age (as in Bernardo and Muñoz 1993), but with model uncertainty. The model has subsequently been extended by Wis´niowski et al. (2015) to include age by applying a common framework, based on the Lee and Carter (1992) approach.

Finally, the existing component forecasts related to UN WPP—Alkema et al. (2011) and Raftery et al. (2013)—as well as the (so far) deterministic assumptions on migration have been combined in the prototype Bayesian population forecasts for the whole world (Raftery et al. 2012; Gerland et al. 2014). These models retain the use of double logistic curves, which the previous work by the UN Population Division has shown to work well in capturing mortality and fertility transitions, but they reduce the need for expert judgement by using formal statistical models to estimate parameters and synthesize multiple data sources. The methods use commonalities between countries to help estimate trends in countries with unreliable or missing data. Moreover, the new methods account for uncertainty in the parameter estimates and future rates, and all estimates and forecasts come with measures of uncertainty. Validation tests suggest that the models are well calibrated and the confidence intervals produced by the models accurately reflect the true level of uncertainty. The computer code to implement the new methods is available through a set of R packages, such as bayesPop, bayesTFR, bayesLife, and bayesDem (for links, see Raftery et al. 2012).

The development of methods for Bayesian forecasting of migration rates for the whole world is currently in progress (Azose and Raftery 2015). The picture of future uncertainties that emerges from the Bayesian methods so far has been quite different from that suggested by the traditional scenario-based projections issued by the UN Population Division. Once the work related to the UN WPP is completed, with well-calibrated assessment of uncertainty stemming from all three components of population change, this will become a very significant step towards including Bayesian modelling in the methodological state of the art of population forecasting.

### 3.2. Limited data

Besides forecasting, Bayesian methods are also very well suited to two types of limited data frequently encountered in demography: data that are sparse and data that are unreliable or incomplete.

Demographers often work with data sets, such as censuses or vital registration data, which are orders of magnitude larger than the data sets used by other social scientists. However, the ever-increasing demand for disaggregated statistics, such as life tables for small areas, or fertility schedules for ethnic groups, means that demographers often deal with sparse data. In other words, once events have been cross-classified along multiple dimensions such as age, sex, region, time, and population size, the number of events within each cell or the number of person-years of exposure can be small. In a small country, it is common to encounter small cell sizes even when working at the national level. In New Zealand, for instance, it is common for annual deaths of five-year-olds to equal zero. Small numbers would pose no methodological problems if, like survey statisticians, demographers were mainly interested in ‘finite population’ quantities, such as the number of deaths that actually occurred. However, demographers are typically interested in ‘super-population’ quantities such as ‘current mortality conditions’ (Vaupel 2002, p. 366). For instance, New Zealand demographers do not conclude that five-year-olds have no chance of dying in years when no five-year-olds in fact die.

One approach traditionally used in demography to analyse sparse data is to combine cells until the number of events within each cell is large enough that random variation no longer dominates. An alternative is to retain detailed classifications but to draw a curve through the observed rates that tries to pick out genuine changes in the underlying rates while filtering out random fluctuations. A curve can be obtained by fitting a low-dimensional parametric model, such as a model life table, or a parametric fertility, mortality, or migration schedule, by ‘graduating’ the rates, or by fitting some sort of general-purpose smoother, such as a spline (Preston et al. 2000; Keyfitz and Caswell 2005). The challenge with all these approaches is finding an appropriate balance between robustness and sensitivity. Aggregations that are appropriate for a small region may be unnecessarily coarse for a large region, for instance, and a spline that works well over most ages may be inappropriately smooth in the young adult groups.

Bayesian hierarchical models provide an elegant solution to the problem of balancing robustness and sensitivity. A hierarchical model is set up with likelihood and prior that pull in opposite directions. The likelihood pulls towards the ‘direct’ estimate, that is, the estimate obtained by simply dividing the observed number of events by the population at risk. The prior pulls towards the predictions from a model for the underlying rates, which, in demographic applications, typically contains a smooth function of age. The posterior for the hierarchical model is, as always, a compromise between likelihood and prior, with the likelihood receiving more weight in cells where there are relatively more observations, and the prior receiving more weight in cells where there are fewer observations. The result is that the hierarchical model is sensitive where it can be, and robust where it needs to be (Gelman and Hill 2006).

**Figure 2:**
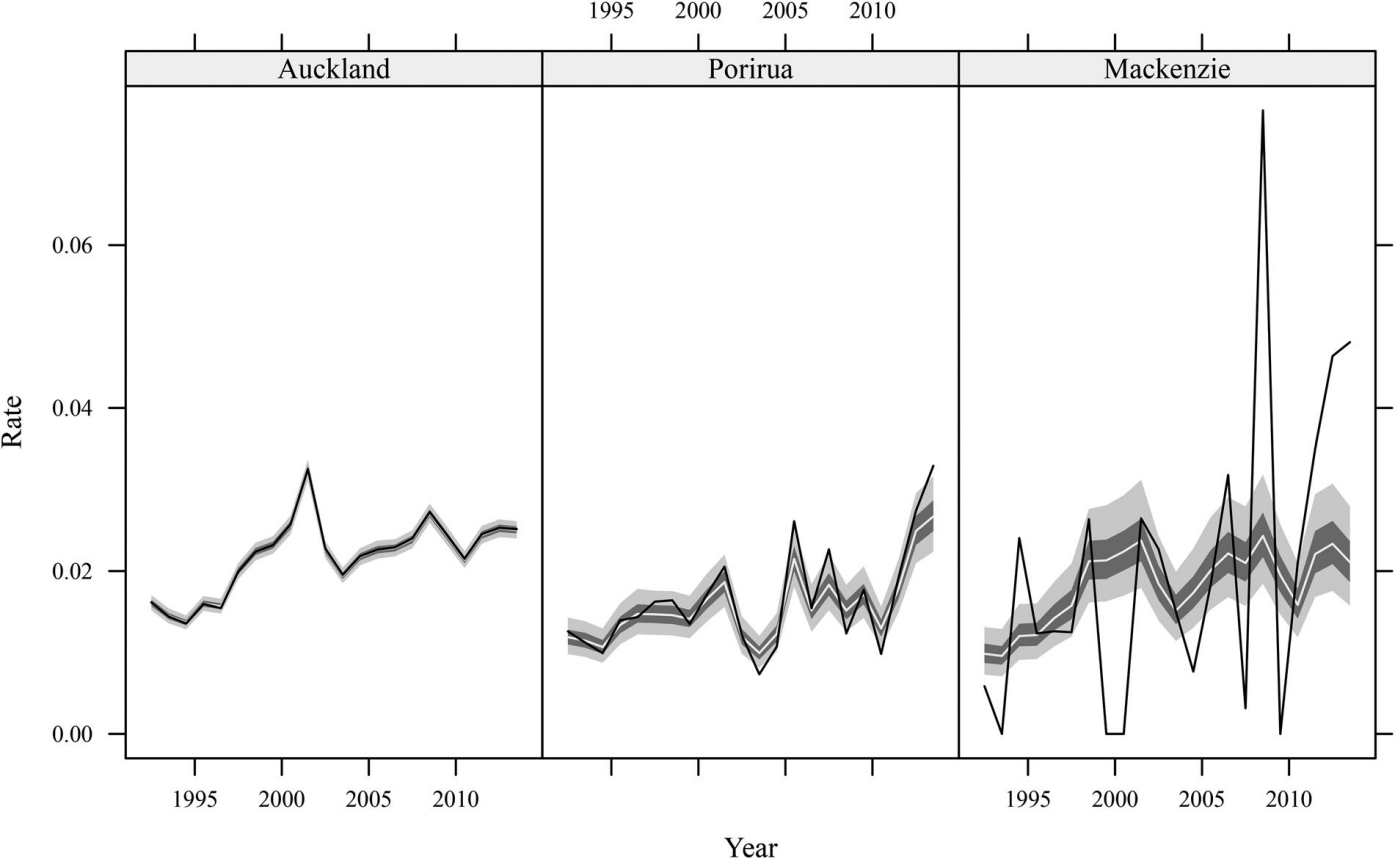
Estimates of annual emigration rates for females aged 30–34 in three selected regions of New Zealand, 1992–2014

*Note*: The light grey bands are 95 per cent credible intervals, the dark grey bands are 50 per cent credible intervals, the pale lines are posterior medians, and the black lines are direct estimates. *Source*: Customized tabulations from Statistics New Zealand.

Figure 2 illustrates these points with estimates of emigration rates from a Bayesian hierarchical model for three New Zealand regions with varying population sizes. The data on numbers of emigrants come from departure cards filled out by everyone entering the country. In 2014, the population of females of Auckland, Porirua, and Mackenzie were 776,900, 27,300, and 2,000. The model gives the most weight to the prior, and the least weight to the direct estimates, in Mackenzie, where the number of observations is smallest and the direct estimates are most erratic. In Auckland, in contrast, the model estimates are almost indistinguishable from the direct estimates.

In recent years, the number of applications of hierarchical Bayes models to demographic questions has been growing rapidly. Representative examples include the following: estimation of marriage rates, including the probability of never marrying, for seventeenth-century Italy (Rosina 2006); a study of geographical variation in mortality in modern Italy (Divino et al. 2009); a model of age at first birth in Nigeria (Gayawan and Adebayo 2013); estimation of mortality rates by migration status in New Zealand (Richardson et al. 2013); estimation of fertility rates in over 5,000 municipalities in Brazil (Schmertmann et al. 2013); and an analysis of intergenerational ‘transmission’ of fertility patterns (Osiewalska 2013). Some Bayesian principles, including heavy reliance on prior information, have been also used by Schmertmann (2012) in designing a calibrated-spline method for estimating the age patterns of fertility.

Finally, if existing data are incomplete or unreliable, the analysts have little choice but to bring in extra information not contained in the data themselves. One example is the IMEM (*Integrated Model of European Migration*) project, which produced a migration flow matrix for 31 European countries (Raymer et al. 2013). Only by incorporating information on accuracy, coverage, and definitions was it possible to produce sensible estimates because the input data varied substantially for all three characteristics. That information did not generally exist in quantitative form. Instead, it was elicited from experts as prior distributions (Wis´niowski et al. 2013). Techniques for eliciting prior distributions that accurately reflect the beliefs of subject-matter experts have been reviewed in O'Hagan et al. (2006). Having a standard method for incorporating extra information of this type is an important strength of Bayesian analysis. The extra information is easier to incorporate in a Bayesian analysis and the transparency of the process is increased, facilitating criticism and replication.

Other applications of Bayesian modelling to problematic data involve, for example: models with constraints on parameters, such as the proportional hazards model applied to breastfeeding durations (McDonald and Prevost 1997); detecting underreporting of births in China by using discrete-time hazard models and change-point regressions (Merli and Raftery 2000); and adjusting published fertility rates for specific subpopulations, such as the US Hispanics (Rendall et al. 2009). In historical demography, Kasakoff et al. (2014) have explored genealogical data from the nineteenth century to disentangle different correlates of individual wealth in the north-eastern United States. As was the case with forecasting, applications of Bayesian methods to estimating demographic parameters and features of different populations are currently developing rapidly in many areas of population sciences.

### 3.3. Highly structured and complex models

The majority of models traditionally used for estimation and prediction are either static or have simple, one-directional dynamics, such as time-series analyses. Real demographic systems, however, typically include feedback loops, constraints, and rates that change over age, time, and space. Data on different components of the system may have to be assembled from different sources, each with its own biases and different levels of completeness. Moreover, demographic systems typically give rise to many types of uncertainty, arising from incomplete knowledge of historical trends or causal mechanisms, or from random variation in disaggregated counts. No model can ever capture all these complexities, but Bayesian methods, and particularly numerical techniques such as MCMC, allow demographers to build models that would be intractable using traditional statistical alternatives.

**Figure 3:**
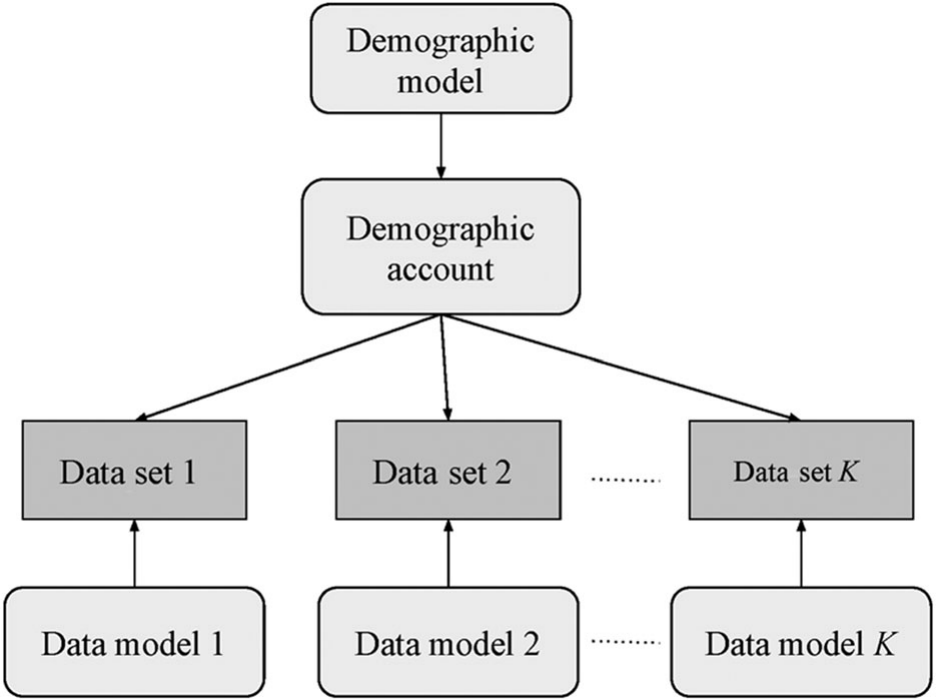
An example of a Bayesian framework for subnational population estimation

*Source*: Adapted from Bryant and Graham (2013, p. 594).

One example of a highly structured model made possible by Bayesian methods is the framework for subnational population estimation presented in Bryant and Graham (2013). The framework is summarized in Figure 3. The dark rectangles in the figure represent known quantities, and the light rectangles represent unknown quantities. Arrows represent probabilistic relationships.

The core of the model is a demographic account (Rees 1979). The account describes all the demographic stocks and flows of interest, linked by accounting identities, and disaggregated by variables such as age, sex, region, and time. In a typical application, the main aim of modelling is to infer values for the demographic account.

Entries within an account typically exhibit strong regularities; for instance, mortality rates have distinctive age profiles, and populous regions tend to stay populous. The overarching ‘demographic model’ captures these regularities. Including the demographic model in the framework means that values within the demographic account that are more demographically plausible receive higher implicit weights. In turn, the individual data models 1, 2, … , *K* capture the relationships between the corresponding *K* data sets and the demographic account. For instance, a data model might state, in mathematical form, that data from the deaths registration system capture 90 per cent of deaths on average, with this relationship varying over age and region.

This framework has some important advantages over more traditional approaches to population estimation. All outputs from the model come with measure of uncertainty. These measures include uncertainty from random variation in demographic events or reporting, uncertainty about demographic rates, and uncertainty about the reliability of the data sources. Because the model ‘predicts’ the contents of each data set from the contents of the demographic account and the corresponding data model, it is easy to deal with missing data, or data that are less detailed than the account: the relevant parts of the demographic account are simply omitted or aggregated before they are supplied to the data model. Some of the most ad hoc and time-consuming parts of the population estimation process are thereby avoided. Because the approach uses statistical models to carry out such tasks as data evaluation that are traditionally accomplished using expert judgement, it is more transparent, and more amenable to replication and automation.

Similar ideas of using Bayesian methods to build complex demographic models for the purpose of reconstructing populations have been discussed by Wheldon et al. (2013, 2016). Their work, illustrated by the example of the populations of Burkina Faso, Laos, Sri Lanka, and New Zealand, is generic and combines the elements of population reconstruction, and a procedure for dealing with missing data. The latter issue is a major problem, especially for many less developed countries.

A second example of highly structured demographic modelling via Bayesian methods is the statistical analysis of fecundity and conception, which involves several structural challenges. Measures such as the number of fertile days per cycle vary from woman to woman, as well as varying over time for the same woman. This variability may be viewed as a noise to be smoothed away, or as an object of interest in itself. Predictors such as frequency of intercourse or daily temperature contain substantial measurement error. Some data are ‘censored’, so that, for instance, the lengths of birth intervals that were still open when recording finished are not known. Fecundity itself follows a complicated non-linear trajectory. Using Bayesian tools such as hierarchical models and MCMC, scholars have made considerable progress in all these areas (e.g., Dunson and Weinberg 2000; Dunson et al. 2002; Dunson and Colombo 2003; McDonald et al. 2011).

A third example of the demographic treatment of complexity is related to computational simulation models that are not tractable analytically owing to their complex structures, presence of non-linear relationships, and possible feedback loops. Here, the associations between model inputs and outputs need to be unravelled. Several approaches have been proposed, including *Bayesian melding* by Poole and Raftery (2000), later extended by Ševčíková et al. (2007), and statistical *emulators—*a special class of *meta-models* of the underlying complex computational models. The emulators are usually based on Gaussian processes, which are also typically analysed within a full Bayesian framework (Kennedy and O'Hagan 2001; Oakley and O'Hagan 2002). A simplified approach also exists—so-called *Bayes linear*—whereby uncertainty beliefs are reflected by the measures of mean and dispersion, rather than by the whole probability distributions (e.g., Vernon et al. 2010).

There are trade-offs between Bayesian melding on the one hand and emulator-based or Bayes linear approaches on the other: the two last-mentioned methods provide only approximate solutions, but may be computationally less expensive to run, especially in the Bayes linear case. Interesting arguments for both approaches can be found, for example, in D. Poole's discussion of Vernon et al. (2010), and the authors’ subsequent rejoinder.

Demographic examples of Bayesian studies of complex computational models include the applications of Bayesian melding in analysing the dynamics of HIV epidemics (Alkema et al. 2007, 2009; Clark et al. 2012; Sharrow et al. 2013), and in agent-based models of transport networks (Ševčíková et al. 2007). Gaussian process emulators have also been used to study an agent-based model of marriage formation (Bijak et al. 2013). Agent-based models are micro-level computational simulation models, whereby the individual units of analysis (*agents*) interact with each other and with their environment according to some rules driving their behaviour. These interactions yield macro-level patterns that can be then compared with the empirical observations (for details, see Billari and Prskawetz 2003). Given the increasing recognition of the complexity of population processes, agent-based models and their analysis using meta-models constitute a very promising path for further enquiries involving the application of Bayesian methods.

## 4. Bayesian demography in the twenty-first century

There are several distinct features of demography that make it especially suited for Bayesian modelling. First of all, the revived interest in uncertainty, and the gradual shift from deterministic to probabilistic perspectives (Alho and Spencer 2005; Courgeau 2012), point naturally to Bayesian methods because of their ability to combine many different uncertainties via probability distributions. Secondly, as discussed above, in applied demography and population statistics there is often a need to combine several data sources, incorporate additional information and constraints, include expert knowledge, and deal with sparse or messy data, all in a coherent manner. Thirdly, as argued by Courgeau (2012), the increasingly popular multilevel paradigm, combining analysis at the levels of individuals, groups, and whole populations, also naturally lends itself to the use of Bayesian methods. This is even truer for the statistical analysis of complex computational models discussed in the previous section.

On the other hand, demography also has a lot to offer to the methodology of Bayesian statistics. First, its strong empirical orientation (Xie 2000; Morgan and Lynch 2001), combined with a uniquely detailed knowledge of some of the underlying mechanisms under study, such as population renewal, can offer a unique testing ground for many Bayesian methods. Second, demographers have already come up with solutions to some specific estimation problems. An example here may be the Lee and Carter (1992) model of mortality surfaces, and its various extensions and revisions, such as those proposed by Girosi and King (2008), who have emphasized the need to preserve good ideas from demography that may be applicable in wider contexts. Third, given its policy relevance, demography can offer statisticians a unique applied area for experimenting with user engagement, communication of uncertainty, and public understanding of statistics. Here, the only coherent framework covering different aspects of user engagement—from uncertain models, estimates, and forecasts, to the informed support of policy decisions and analysis of their possible consequences—is Bayesian.

So, why should more demographers use Bayesian methods, and what would be the value added offered by such approaches to the twenty-first-century population scientists? First, we believe that the notions of risk and uncertainty will probably gain more ground in social science, as an honest and scientifically sound way of describing social reality. A coherent description of uncertainty will become crucial: end users will no longer remain satisfied with point estimates or purely qualitative indications of reliability. Second, even in the age of Big Data, such established techniques as Bayes's theorem will facilitate analysis by helping to avoid false positives, which are one of the dangers of using large-scale data mining techniques. Third, as argued in Section 3, the analysis of complex social phenomena, for example, by using computational computer models, will also need recourse to a formal language that can describe the underlying mechanisms in a coherent, probabilistic fashion. This work is still in its infancy although some promising ideas have already emerged, such as the use of recursive Bayesian networks as a mathematical language for describing causal mechanistic modelling (Casini et al. 2011).

Recent rapid developments in Bayesian demography provide grounds for optimism. As argued by Courgeau (2012), the history of demography and population studies in the past have been largely cumulative, with new paradigms or perspectives, such as longitudinal, event-history, or multilevel modelling supplementing old ones, rather than replacing them completely. We believe there are several important reasons why this cumulativity constitutes a very strong case for furthering the Bayesian perspective in twenty-first-century demography.

Most importantly, as discussed above, the Bayesian platform can be seen as a more general framework encompassing some other probabilistic approaches, especially the purely data-based and purely expert-based ones, in a coherent manner. As such, Bayesian approaches are complementary to many traditional demographic methods, rather than being in direct competition with them, since these traditional methods can be productively re-expressed in Bayesian terms. The very essence of Bayesian inference is based on the notion of continuity—constant updating of beliefs in the light of new evidence, in line with the main tenets of the scientific method. It allows incorporating new insights—quantitative data, as well as expert views, some of which may be qualitative—rather than reinventing the existing knowledge base or, worse, ignoring it. The theory and methods, as well as the logical and philosophical underpinnings of Bayesian statistics are also continually developing, partially in response to various contemporary scientific challenges (for a discussion, see, e.g., Williamson and Corfield 2002).

There are several challenges for the further developments of Bayesian demography, and for its practical applications. In our view, three of them are of special importance. Firstly, there is a lack of training in Bayesian methods at the undergraduate and postgraduate levels in the social science and statistics curricula. For Bayesian demography to gain further momentum, more training opportunities—some of which already exist, especially in the form of elective courses—should be offered to practising demographers and social statisticians. Second, current computational methods are mainly targeted at academic users, rather than practitioners: although, as discussed above, there are few general-purpose platforms, many Bayesian solutions are bespoke. Until recently there was a distinct lack of Bayesian modules in mainstream software, with the current exception of R (Park 2015) and SAS (SAS Inc., n.d.).

A separate, important challenge is the communication of uncertainty to the users so that they can make the most of the information provided to them via probability distributions (Bijak et al. 2015). This problem is not limited to demographic or social science applications, and there is already considerable work in this area more broadly (e.g., Spiegelhalter et al. 2011). A related issue is connected to the practical utility of the probabilistic outcomes of population estimates and forecasts. A promising practical extension of Bayesian estimation and forecasting consists of a formal decision analysis, introduced briefly in Section 2.3, which could be used for policy and planning purposes to mitigate the expected uncertain outcomes.

Contemporary applied Bayesian statistics does not emphasize the connection with the decision analysis, and one does not have to commit to utility theory to be a Bayesian—for example, prominent orthodox Bayesians such as Lindley (1992) argue that the main outcomes of any Bayesian analysis are whole distributions rather than point estimates. However, applications of Bayesian decision theory have been suggested to solve practical problems, including demographic ones. This approach could help select appropriate values from the probability distributions, which could be then used for policy or planning purposes (see Alho and Spencer 2005; Bijak 2010). The main methodological challenge here is the elicitation of the utility or loss function from the users of the analysis. Hence, despite the scarcity of concrete applications so far, the framework is there if it is needed, and in our view this is one of the important directions for the practice of applied Bayesian demography in the future.

We think that these features of Bayesian statistics—and Bayesian demography—are really remarkable for a 250-year-old invention, and that they bear promise of many further exciting developments in the applied population sciences throughout the twenty-first century.
